# Exploring the Promise of Endophytic Fungi: A Review of Novel Antimicrobial Compounds

**DOI:** 10.3390/microorganisms10101990

**Published:** 2022-10-08

**Authors:** Daniel J. Caruso, Enzo A. Palombo, Simon E. Moulton, Bita Zaferanloo

**Affiliations:** 1 Department of Chemistry and Biotechnology, School of Science, Computing and Engineering Technologies, Swinburne University of Technology, Hawthorn, VIC 3122, Australia; 2 Department of Engineering Technologies, School of Science, Computing and Engineering Technologies, Swinburne University of Technology, Hawthorn, VIC 3122, Australia

**Keywords:** endophytes, bioactive compounds, antimicrobial, antibiofilm, natural products, novel, antimicrobial resistance

## Abstract

Over the last few decades, many of the existing drugs used to treat infectious diseases have become increasingly ineffective due to the global emergence of antimicrobial resistance (AMR). As such, there is a constant demand to find new, effective compounds that could help to alleviate some of this pressure. Endophytic fungi have captured the attention of many researchers in this field, as they have displayed a vast ability to produce novel bioactive compounds, many of which possess wide-ranging antimicrobial activities. However, while highly promising, research in this area is still in its infancy. Endophytes inhabit the healthy tissues of plants asymptomatically, resulting in a mutualistic symbiosis in which the endophytes produce a plethora of bioactive compounds that support the fitness of the host plant. These compounds display great chemical diversity, representing structural groups, such as aliphatic compounds, alkaloids, peptides, phenolics, polyketides and terpenoids. In this review, the significant antimicrobial potential of endophytic fungi is detailed, highlighting their ability to produce novel and diverse antimicrobial compounds active against human, plant and marine pathogens. In doing so, it also highlights the significant contributions that endophytic fungi can make in our battle against AMR, thus providing the motivation to increase efforts in the search for new and effective antimicrobial drugs.

## 1. Introduction

One of the most pressing issues facing humanity today is the threat posed by infectious diseases. The emergence of drug-resistant pathogenic microorganisms over recent decades has raised further challenges in terms of public health and well-being, while the emergence of new and deadly viruses has further complicated matters. Perhaps there is no greater example of this than the current COVID-19 pandemic, caused by an outbreak of a new coronavirus that has so far infected more than 600 million people and resulted in the deaths of more than 6.5 million people around the globe [1]. Infectious disease control efforts in farming and agriculture have also contributed to serious environmental issues due to the use of toxic insecticides and other compounds, which inevitably end up in the environment, leading to problems such as an increased loss of biodiversity and pollution of both terrestrial and aquatic environments [2,3]. To address some of these problems, there is a constant demand to find new and effective bioactive compounds that can help to alleviate some of the burden associated with the use of toxic, synthetic and ineffective compounds. Naturally occurring bioactive compounds, or natural products, are ubiquitous in nature, often possessing great and wide-ranging bioactivities, as well as being less toxic and more environmentally friendly [4]. Traditional sources of natural products include plants, microorganisms and animals. More recently, however, advancements in technologies led the search for new bioactive compounds away from those of natural origins towards the screening of large chemical libraries composed of synthetic molecules. While these high-throughput and automated methods may have seemed promising, such an approach has seen little success. As a result, interest in natural metabolites as a source of new bioactive compounds is again on the rise. Many plants and microorganisms have been studied, providing a wealth of new drug molecules, particularly those with anticancer and anti-infective properties [4,5,6]. In recent times, one group of largely unexplored microorganisms has increasingly become the focus of such investigations, that being endophytes. Endophytes are microorganisms, fungi and bacteria that inhabit the tissues of healthy plants without causing any symptoms of harm or disease. They exist in a symbiotic relationship with their host plant, in which they have been shown to produce a vast array of bioactive compounds that contribute to the overall fitness and survival of the plant. Many of these compounds have been found to possess bioactivities that have potential applications in medicine, agriculture and industry [7,8]. Endophytes have been isolated from all plants studied to date, and it is widely believed that each of the some 300,000 species of plants that exist on Earth are host to at least one endophytic resident, yet only around 1–2% of plant species have been studied in relation to their endophyte populations [7]. Even so, the diversity of the chemical compounds so far discovered from endophytes is vast, both in terms of structure and bioactivities. Furthermore, some estimates suggest there may be as many as 1 million different species of endophytic fungi, the large majority of which are yet to be described [9]. Hence, endophytic microorganisms represent a highly promising source for the discovery of novel natural substances.

This section will provide some background and history of natural products research, as well as that of endophytes, including their ecological role, classification and some of the rationale involved in choosing a plant for study.

### 1.1. Natural Products

Natural products are compounds biosynthesised by living organisms, including plants, microorganisms and animals. Most often, the term refers to secondary metabolites, which differ from primary metabolites in that they are unnecessary for growth or reproduction. Instead, they may be produced as the result of environmental adaptions or perhaps as a means of deterring predators, in either case, aiding in the survival of the organism [10]. Secondary metabolites are generally small organic molecules characterised by their low molecular weight, as well as their diverse chemical structures and biological activities [11]. Such compounds have had a tremendous impact on modern medicine, highlighted by the fact that approximately 42% of the new drugs approved during the last 40 years are either natural products or their derivatives, while over 52% of the anti-infective small molecule drugs approved during the same time frame can be attributed to natural sources [12]. Human use of natural substances dates back much further than this; however, the earliest known records originate from the ancient Sumerians almost 5000 years ago [13]. Throughout history, cultures all around the world developed their own pharmacopoeias based on the natural resources available to them. The Ebers papyrus is a compilation of Egyptian medical texts written around 1550 B.C., describing the use of more than 700 plant-based drugs against a wide range of afflictions. The Materia Medica, written around 1100 B.C., is one of several medical documents detailing the use of natural medicines in China. This, along with the Shennong Herbal from 100 B.C. and the Tang Herbal from 659 A.D., describe the uses of hundreds of different natural remedies. The Greeks and the Arabs each had their own wealth of knowledge regarding the use of a range of medicinal herbs [13], while the Charaka Samhita provides the most comprehensive description of the traditional Indian Ayurvedic medical system, including details of hundreds of medicinal plants and their uses [14].

While plants have served as the basis for the vast majority of traditional medicines, microorganisms have also proven to be a valuable source of bioactive secondary metabolites. The so-called Golden Age of Antibiotics began in 1929 when Alexander Fleming discovered the penicillin-producing fungus *Penicillium notatum* [15], leading to the isolation of penicillin a decade later by Howard Florey’s team [16]. Investigations into the microbial sources of bioactive molecules increased significantly following this success, leading to the discovery of numerous antibiotics, such as the cephalosporins from the fungus *Cephalosporium acremonium*, as well as the aminoglycosides, tetracyclines and chloramphenicol, each of which was isolated from various species of *Streptomyces* bacteria [17]. The glycopeptide antibiotic, vancomycin, was first isolated in 1953 from cultures of the bacterium *Amycolatopsis orientalis*, while erythromycin, the progenitor macrolide and a metabolite of the bacterium *Saccharopolyspora erythraea*, was also discovered during the 1950s [13,18]. The impact of microbial metabolites on the pharmaceutical industry is not only limited to antibiotics. Some of the most successful examples include cyclosporins and rapamycin, immunosuppressant compounds that were originally isolated from the fungus *Tolypocladium inflatum* and the bacterium *Streptomyces hygroscopicus*, respectively [15]. Development of the cholesterol-lowering statins began with the discovery of mevastatin (compactin), a metabolite of the fungus *Penicillium citrinum*, in 1973, and lovastatin, produced by the fungus *Aspergillus terreus*, in 1979, commercialisation of which was followed by the introduction of a further two semi-synthetic and four synthetic varieties, resulting in the largest selling class of drugs worldwide, with sales amounting to 25 billion in 2005 [19]. The discovery of avermectin from the fermentation broth of the bacterium *Streptomyces avermectinium* led to the development of a new class of potent antihelmintic and antiparasitic drugs, such as the semi-synthetic derivative ivermectin, which are used to treat a broad range of worm and other parasite-related diseases [20]. The researchers involved in the discovery of avermectin were awarded the 2015 Nobel Prize in Physiology or Medicine, while in 1945 and 1952, the same award went to those involved in the discovery of penicillin and streptomycin (an aminoglycoside), respectively, highlighting the enormous impact that natural microbial products have had on modern medicine.

Natural products have undoubtedly had an enormous impact on human health and well-being, both historically and in the modern context. However, despite this success, research into natural compounds as a source of new drugs steadily declined over recent decades. Technological developments allowed for much greater automation, which enabled a high-throughput screening approach to the drug discovery process, quickly becoming the preferred method within the pharmaceutical industry. Combinatorial chemistry techniques provided the means to produce large libraries of synthetic compounds, while developments in cellular and molecular biology, as well as genomics, led to the discovery of many new molecular targets. As a result, screening vast libraries of synthetic compounds against a specific biological target became possible, leading industries to eventually abandon much of their research on natural substances in favour of such an approach [21]. There has, however, been very little success using combinatorial methods [12], owing somewhat to the fact that the compounds produced are significantly less diverse, with lower degrees of chirality and steric complexity relative to the compounds produced by nature [21]. In contrast, natural products have great structural diversity and are the result of evolutionary processes. They are, therefore, highly adapted for biological functions, acting on biochemical pathways with a great deal of specificity and with a high affinity for their particular target receptors [21]. Furthermore, especially with regards to microorganisms, the understanding of the biochemical pathways involved in the synthesis of secondary metabolites has progressed to a point where targeted modification and manipulation is now a possibility, while genomic insights have revealed that the genes involved in their biosynthesis are capable of producing a much greater diversity of compounds than was ever previously imagined [22]. Considering their favourable properties, along with the growing insight into their biochemistry and biosynthesis, the advantages of natural products in the drug discovery process are again becoming clear.

### 1.2. Endophytes

Anton de Bary introduced the term endophyte in 1866, a contraction of the Greek “*endon*”, meaning within, and “*phyton*”, meaning plant [23]. The term in itself thus provides the most basic definition in that it refers to any organism living within a plant, in contrast to those which live on the outside of plants, referred to as epiphytes (Gr. “*epi*” meaning upon or on top of). However, such a definition is quite vague and lacks sufficient meaning. While more recent attempts to define endophytes have been the subject of varying degrees of criticism for their apparent lack of consistency and wider acceptance [24,25,26], endophytes today are generally regarded as any organism which occupies healthy plant tissues asymptomatically [27]. More explicitly, the term refers to certain microorganisms, specifically fungi and bacteria, that can, for all or part of their life cycle, colonize both the inter- and intra-cellular spaces of various plant tissues without causing any sign of harm or disease [28]. Fossil records indicate that such associations between plants and fungi are ancient, dating back some 400 million years to around the same time as terrestrial plants began to emerge [29]. It is, therefore, no surprise that through this co-evolution, the symbiotic relationship that has developed between plants and their endophytic inhabitants is one of great complexity and importance. In fact, it is believed that each of the 300,000 species of plants existing on Earth is host to at least one or more fungal endophytes, yet only a handful of plants have been studied in relation to their endophytes to date [30].

Endophytes appear to be critical to plant health and function and, by extension, play a crucial role in wider ecosystems. For example, endophytes are known to enhance plant growth through a variety of mechanisms, including the production of phytohormones, such as indole-3-acetic acid (IAA) [31] and gibberellins [32], the production of siderophores [33], as well as compounds which are able to increase solubilisation and availability of inorganic potassium, phosphorus and other elemental nutrients [34,35]. The host plant’s ability to tolerate a range of biotic and abiotic stress factors is also increased due to the presence of endophytes. For example, tolerance to abiotic stress factors, including high temperatures [36,37,38,39,40], low temperatures [41,42], high salinity [36], drought [37,39,40] and heavy metals [40,43] is enhanced by endophytes. Similarly, endophytes also play an important role in enhancing the ability of a host plant to resist biotic stress factors, such as disease [44,45] and herbivores [46]. Colonisation by endophytes can increase plant resistance to microbial pathogens by several different mechanisms, including through the production of antimicrobial metabolites [47,48], creating physical and chemical barriers serving as plant defence components [49,50], up-regulation of genes involved in host defence responses [51], induction of systemic resistance [52,53] and by outcompeting pathogens for space [54]. Deterrence of herbivory due to the production of toxic alkaloids by endophytes is well documented [55,56], while endophytic protection against oomycetes [57] and parasitic nematodes [58] have also been reported. The ability of endophytes to effectively mediate such factors has been comprehensively reviewed in the literature [59,60,61].

### 1.3. Classification of Endophytic Fungi

In contrast to mycorrhizal fungi, which form associations with plant roots, growing into the rhizosphere, endophytic fungi are contained entirely within the tissues of plant roots, stems and leaves, sporulating once a plant or the host tissue begins the process of senescence [62,63]. Fungal endophytes have been designated as belonging to one of two groups; the clavicipitaceous endophytes (C-endophytes) that infect cool- and warm-season grasses and the non-clavicipitaceous endophytes (NC-endophytes) that occupy vascular and non-vascular plants [64]. Each group is defined according to criteria, such as their host range, the types of tissue they colonise, their degrees of colonisation and diversity *in planta*, their mode of transmission and how they benefit their host plant [62]. The C-endophytes, referred to as Class 1 endophytes, are characterised by their narrow host range, lower phylogenetic diversity, extensive colonisation within the plant, transmission through both vertical and horizontal modes and the nonhabitat-adapted fitness benefits they provide to the host plant. On the other hand, the NC-endophytes consist mainly of ascomycetous fungi and are further divided into three different classes (Class 2, 3 and 4). While all three classes of NC-endophytes have a broad host range, Class 2 can colonise both above- and below-ground tissues of the shoot, root and rhizome, whereas Class 3 and Class 4 can only colonise the above-ground tissues of the shoot and root, respectively. Limited colonisation *in planta* is a feature of Class 3, while Class 2 and Class 4 are capable of extensive colonisation. Class 2 and Class 3 often have a low and high diversity, respectively, within a given host plant, while that of Class 4 is not currently known. Class 2 transmits both vertically and horizontally, whereas Class 3 and Class 4 each transmit solely through the horizontal mode. The final point of difference is that, like Class 1, each of the other three classes provides nonhabitat-adapted fitness benefits; however, Class 2 is unique in the fact that they can provide habitat-adapted fitness benefits based on habitat-specific pressures, including the pH, temperature and salinity [62]. Furthermore, the NC-endophytes occupy all major lineages of land plants, representing all terrestrial ecosystems from the tropics to the tundra [63,64].

### 1.4. Plant Selection

The population of endophytes not only varies between plants and species but also within the same species, based on the differences in location as well as climatic and local environmental conditions [65]. Given such variations and considering the great number of plant species in the world, understanding the rationale and methods applied when selecting a plant for endophyte investigation is important, as this can maximise the probability of discovering novel and useful bioactive compounds. In studies aimed at isolating endophytes for the purposes of natural product discovery, there are several approaches to this selection. Firstly, plants that grow in unique environments, particularly if those plants have unusual biology or a novel survival strategy. For example, Singh et al. (2020) [66] isolated the endophytic bacterium *Bacillus amyloliquefaciens* 5NPA-1 from *Salicornia brachiata*, a halophytic plant that grows in salty marsh lands, collected from the Gujarat coast, India. Antimicrobial screening of the *B. amyloliquefaciens* 5NPA-1 extract revealed strong inhibitory action against the human pathogen *Staphylococcus aureus* and the phytopathogen *Xanthomonas campestris* [66]. Secondly, plants that are endemic to a region, especially in areas which were once part of an ancient land mass or those with an unusually long lifespan, can be considered. Thirdly, plants growing in areas of high biodiversities, such as tropical rainforests, are also expected to harbour an equally biodiverse range of endophytes. Finally, plants with a history of ethnobotanical use relating to the intended application of a specific investigation can be considered.

Plants with a history of medicinal use have been the focus of many endophytic studies, owing to the fact that the medicinal properties of the selected plant may stem from the endophytic residents rather than the plant itself. This line of thought began with the discovery of a taxol-producing endophytic fungus, *Taxomyces andreanae*, isolated from the inner bark of the Pacific yew tree (*Taxus brevifolia*) [67]. Taxol is a highly functionalised diterpenoid that is used in the treatment of various cancers, originally isolated from the bark of the Pacific yew. The discovery of the taxol-producing *T. andreanae* suggested that the endophytic fungus was the actual source of taxol isolated from the tree or that it was at least able to produce the same compound as its host plant. The realisation that the medicinal properties of plants could be related to their endophytic populations was also the rationale followed that led to the discovery of munumbicins, wide-spectrum antibiotic peptides active against a range of human and plant-pathogenic fungi and bacteria, as well the malarial parasite *Plasmodium falciparum* [68]. The munumbicins were isolated from a novel endophyte, *Streptomyces* sp. strain NRRL 30562, obtained from snakevine (*Kennedia nigriscans*), a medicinal plant native to the Northern Territory of Australia. Snakevine was selected for study on the basis of having been used by local Aborigines, who would crush and heat the plant in an aqueous brew to treat cuts, wounds and infections [68]. Hence, it is reasonable to assume that the therapeutic properties associated with the plant, known to the local indigenous peoples, may be the result of compounds produced by not only the plant but also the specific endophytes associated with it.

## 2. Antimicrobial Potential of Endophytic Fungi

Recent years have seen the number of publications relating to endophytes and their bioactive products steadily rise, with many reviews detailing a diverse range of bioactive compounds. As an example, the antitumor compounds isolated from a range of endophytic fungi in the years between 2010 and 2013 were described in a review by Chen et al. (2016) [69]. A more recent review is that by Nalini and Prakash (2017) [70], in which the authors detailed numerous compounds isolated from filamentous actinomycete bacterial endophytes which possessed a range of bioactive properties, such as antibiotic, antioxidant, cytotoxic, antitumor, antimalarial, anti-filarial and enzyme-inhibition [70]. A systematic review of the literature by Raimi and Adeleke (2021) [71] provides great detail regarding the therapeutically important bioactive compounds isolated from endophytes between 2000–2019, including many with antibacterial, antifungal and anticancer activities [71].

The following section is focused on the antimicrobial compounds that have been isolated from endophytic fungi in recent times. Firstly, a range of crude extracts obtained from endophyte cultures that were reported to display in vitro antimicrobial activities is described. A summary of these antimicrobial extracts is provided in Table 1. Secondly, several examples of identified endophyte-produced antimicrobial compounds, representing the various chemical classes that are most frequently isolated, are also described, along with the antimicrobial properties reported for each. These antimicrobial compounds are summarised in Table 2. Finally, a number of metabolites isolated from endophytes have shown antibiofilm activities. These are also discussed below.

### 2.1. Endophyte Culture Extracts with Antimicrobial Properties

In general, bioprospecting of endophytes for the production of antimicrobial metabolites begins with a relatively simple screening process which is used to identify promising candidate strains for further analysis. To do so, endophytes are grown in either solid or liquid cultures from which a crude organic or aqueous extract can be obtained. These extracts can then be screened for the desired activity against the chosen test organisms by using rapid techniques such as a disk diffusion or well diffusion assay. For active extracts, the minimum inhibitory concentration (MIC) provides a more quantitative measure of the activity, which is easily determined using a broth dilution assay or a similar method. These methods provide a simple, fast and inexpensive way to evaluate numerous extracts in a short amount of time.

Two endophytic fungi, *Papulaspora immersa* and *Arthrinium* state of *Apiospora montagnei* Sacc., were described by Ramos et al. (2010) [72]. Various extracts of these endophytes, isolated from *Smallanthus sonchifolius* collected in Brazil, each displayed antibacterial activity against a range of test organisms. For *P. immersa*, the ethyl acetate extract of the fermentation broth filtrate was found to inhibit the growth of *Pseudomonas aeruginosa*, *S. aureus* and *Kocuria rhizophila*, while *n*-butanol and the aqueous extracts of the same filtrate were effective against *S. aureus* and *Escherichia coli*. The ethyl acetate and *n*-butanol extracts of the filtrate were obtained from the fermentation broth of the *Arthrinium* state of *A. montagnei* Sacc. and were each active against *E. coli* and *P. aeruginosa* [72].

A novel endophytic fungus, isolated from the healthy leaves of *Vitex negundo* L., a medicinal plant collected in India, was reported by Arivudainambi et al. (2011) [73]. The isolated fungal endophyte was identified as *Colletotrichum gloeosporioides* based on morphological and molecular observations. The methanolic extract obtained from the filtrate of the *C. gloeosporioides* fermentation broth displayed antibacterial activity against a range of both Gram-positive and Gram-negative species, including *S. aureus*, *Bacillus subtilis*, *E. coli* and *P. aeruginosa*, as well as antifungal activity against the fungal pathogen *Candida albicans*. Several strains of methicillin-resistant *S. aureus* (MRSA) were also found to be susceptible to the same methanolic extract, for which MIC values in the range of 31.25–250 µg mL^−1^ were reported. Furthermore, when the methanolic extract was used in combination with the antibiotics penicillin and vancomycin, the authors reported synergistic effects that resulted in significant decreases in the MIC values of each antibiotic [73].

In the same year, a number of endophytic fungi were isolated from the Vietnamese plants, *Dendrobium devonianum* and *Dendrobium thyrsiflorum*, by Xing et al. (2011) [74]. Morphological characteristics and molecular analysis were used to identify isolates, with the majority belonging to the genera *Fusarium*, *Phoma* and *Epicoccum*. The ethanolic extracts obtained from the fermentation broth filtrates of several of these endophytic isolates displayed significant inhibitory activity against a range of bacterial and fungal test species, comparable in some cases to those displayed by antibiotic controls. Extracts from the endophytic isolates *Phoma* sp., *Epicoccum nigrum* and *Fusarium tricinctum* reportedly produced the largest inhibition zones against *B. subtilis* (21.6 ± 0.7 mm) and *E. coli* (20.8 ± 0.7 mm), *S. aureus* (20.2 ± 0.3 mm) and *C. albicans* (15.5 ± 0.5 mm), respectively [74].

Pretsch et al. (2014) [75] reported the antimicrobial activity of *Talaromyces wortmannii*, a fungal endophyte isolated from an Egyptian *Aloe vera* plant. Significant antibacterial activity was observed when the ethyl acetate extract of a solid-state culture of *T. wortmannii* was tested against a number of Gram-positive strains, including *Propionibacterium acnes*, *Staphylococcus epidermidis*, *Enterococcus faecalis*, MRSA and *Streptococcus pneumoniae*, with MIC values of 3.9, 7.8, 7.8, 15.7 and 31.5 µg mL^−1^, respectively, whilst Gram-negative bacteria were found to be less susceptible, with MIC values ranging from 62.5–125 µg mL^−1^. Subsequent fractionation of the crude extract resulted in significantly lower MIC values of 0.24, 0.98 and 1.90 µg mL^−1^ against *S. epidermidis*, *P. acnes* and MRSA, respectively, matching or even outperforming the activity of the standard antibiotics chloramphenicol, ampicillin and vancomycin, which had MIC values in the range of 0.98–3.91, 0.98–3.91 and 1.98–3.91 µg mL^−1^, respectively [75].

In that same year, Yang et al. (2014) [76] isolated several fungal endophytes from the stem and root tissues of *Anemone tomentosa*, a perennial herb from China. Both ethyl acetate and *n*-butanol extracts obtained from the liquid culture filtrate of an unidentified root-associated endophyte displayed wide-spectrum antibacterial activity, with a MIC value of 0.156 mg mL^−1^ recorded against Gram-positive species, including *S. aureus*, *Bacillus licheniformis* and *Streptococcus uberis*, as well as for Gram-negative species, including *E. coli*, *P. aeruginosa* and *Klebsiella pneumoniae*. Similar MIC values for the methanolic mycelial extract obtained from a second unidentified root-associated fungal endophyte were observed, with values ranging from 0.156–0.313 mg mL^−1^ against the same test organisms [76]. These MIC values, however, correspond to the lowest concentration used during testing, meaning the true MIC could potentially be lower than those reported.

Several fungal endophytes, including *Alternaria* sp., *Bjerkandera* sp., *Diaporthe* sp., *Penicillium* sp. and *Xylaria* sp., were obtained from the leaves of *Schinus terebinthifolius* by Tonial et al. (2016) [77]. The ethyl acetate extracts that were prepared from culture filtrates of the various endophytic fungi displayed antibacterial activity against *S. aureus* and *P. aeruginosa*, as well as antifungal activity against *C. albicans*, as did the methanolic extracts of their mycelium. The fermentation conditions of three isolates, including *Alternaria alternata*, *Bjerkandera* sp. and *Xylaria* sp., were subsequently investigated to determine the optimal conditions for antimicrobial production, with various parameters, such as different carbon sources, nitrogen sources, pH, temperature and incubation times all being explored. The authors reported that the response to changes in these various conditions differed between endophytic strains, while antimicrobial activity was generally at its highest when the carbon source was galactose [77]. Such findings highlight the importance of the conditions during the culturing of endophytes and the effects this can have on metabolite production.

An endophytic fungus, *Aspergillus clavatonanicus* strain MJ31, was isolated by Mishra et al. (2017) [78] from root tissues of the Indian plant *Mirabilis jalapa* L. A combination of morphological characteristics and molecular data, including the sequencing of the rRNA ITS region, 28S rRNA and the translation elongation factor 1- alpha (EF 1α) genes, were used to identify the specimen. An ethyl acetate extract obtained from the filtrate of the *A. clavatonanicus* fermentation broth displayed antibacterial activity against both Gram-positive and Gram-negative species, including *B. subtilis*, *Micrococcus luteus*, *S. aureus* and *E. coli*, with MIC values of 0.078, 0.156, 0.312 and 0.625 mg mL^−1^, respectively. Interestingly, seven known antibiotics were also reportedly detected and quantified from the same ethyl acetate extract, including ampicillin, chloramphenicol, fluconazole, ketoconazole, miconazole, rifampicin and streptomycin, with concentrations ranging from 30 µg g^−1^ for streptomycin, up to 900 µg g^−1^ in the case of miconazole [78].

In the same year, Atiphasaworn et al. (2017) [79] reported the isolation of several fungal endophytes belonging to eight different genera from the leaves of the *Ocimum basilicum* var. *thyrsiflora* collected in Thailand. All isolates were identified by a combination of morphological observations and sequence analyses of the ITS region of rRNA. Ethyl acetate extracts of the mycelium and fermentation broth of each specimen displayed some level of antibacterial activity against a range of nine human pathogenic bacteria, with each extract generally showing selectivity towards either Gram-positive or Gram-negative species. However, one endophytic isolate, *Nigrospora* sp., displayed a broad spectrum of activity, with significant results observed against *E. coli*, *S. epidermidis*, *Vibrio cholerae* and *Bacillus cereus* with MIC values of 7.81, 62.5, 125 and 250 µg mL^−1^, respectively, on par with the standard antibiotic chloramphenicol in each case [79].

In 2019, Ikram et al. [80] described the isolation of two fungal endophytes from the roots of *Solanum surattense*, a medicinal plant collected from salt marshes in Pakistan. The two isolates were identified as *Penicillium roqueforti* and *Trichoderma reesei* based on both their morphological characteristics and the sequencing of the ITS region of rDNA. The ethyl acetate extracts obtained from the culture supernatant of each isolate exhibited significant inhibitory activity against several plant-pathogenic bacteria. Of the two, the *P. roqueforti* extract showed the greatest activity, with MIC values of 0.625, 1.25, 5 and 10 µg mL^−1^, and minimum bactericidal concentrations (MBC) of 1.25, 2.5, 10 and 10 µg mL^−1^ reported against *Agrobacterium tumefaciens*, *Xanthomonas oryzae*, *Pseudomonas syringae* and *Ralstonia solanacearum*, respectively. In the case of *T. reesei*, the MIC values were largely the same as those reported for *P. roqueforti*, while the MBC values were generally higher [80].

In 2021, numerous endophytic fungi were isolated from various parts of *Bruguiera sexangula*, a mangrove species collected from Hainan Island in China [81]. Both ascomycetes and basidomycetes were isolated, although the ascomycetes were the dominant phylum, with representatives of several genera, including *Fusarium*, *Gelasinospora*, *Diaporthe* and *Pestalotiopsis*, amongst others. The ethyl acetate extracts of several isolates grown on four different media were screened for antimicrobial activity against various pathogens, whereas the isolate, *Gelasinospora endodonta*, grown on Czapek’s medium, displayed significant inhibitory activity against the Gram-negative *E. coli*, with a MIC of 0.0625 mg mL^−1^. When this isolate was grown on potato dextrose broth (PDB), the MIC against *E. coli* was significantly higher at 1 mg mL^−1^, while extracts from the same isolate cultured on rice medium and grain medium were both found to be inactive [81]. Such results highlight the importance of the choice of culture media and the impact this can have on the production of antimicrobial compounds by endophytic fungi.

### 2.2. Antimicrobial Compounds of Different Chemical Classes Produced by Endophyte

Endophytes have been reported to produce many bioactive secondary metabolites with antimicrobial activity against a wide range of pathogenic microorganisms. These compounds show great structural diversity and complexity, representing a variety of different chemical classes, such as aliphatic compounds, alkaloids and other nitrogenous compounds, peptides, phenolics, polyketides and terpenoids [82,83]. In the following section, several examples of previously unknown compounds with antimicrobial activity that were first discovered as endophyte metabolites are described according to their broad chemical classification, along with their reported activities.

#### 2.2.1. Aliphatic Compounds

**Figure 1:**
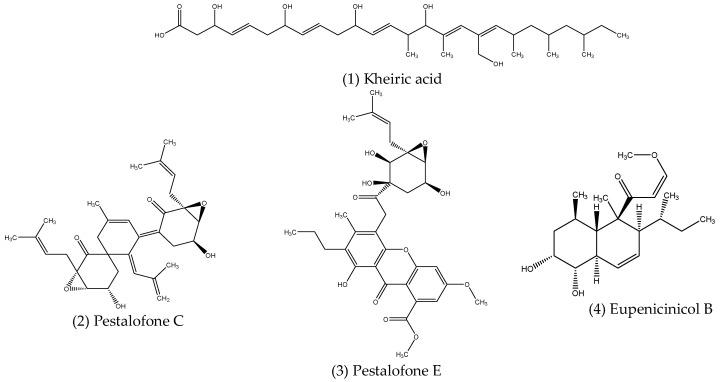
Structures of antimicrobial aliphatic compounds produced by endophytes.

Aliphatic compounds are a class of hydrocarbons consisting of non-aromatic straight or branched chains of mainly carbon and hydrogen atoms, although other elements, such as oxygen, sulphur and chlorine, may also be present. Several aliphatic compounds exhibiting antimicrobial activity have been reported from endophytes, the structures of which are shown in Figure 1 below.

A new polyhydroxy acid named kheiric acid (1) was obtained from *Curvularia papendorfii*, an endophytic fungus isolated from the Sudanese medicinal plant *Vernonia amygdalina*, which displayed antibacterial activity against a strain of MRSA, with a MIC of 62.5 µg mL^−1^ [84]. The endophytic fungus, *Pestalotiopsis fici*, was found to produce pestalofones A (2) and C (3), two new cyclohexanone derivatives which possess significant antifungal activities against *Aspergillus fumigatus* (ATCC 10894), with MIC values of 35.3 and 31.2 µM, respectively [85]. Both pestalofones A and C were active at concentrations much lower than the positive control, fluconazole, which had a MIC of 163.4 µM [85]. Eupenicinicol B (4), a new aliphatic metabolite isolated from the culture of *Eupenicillium* sp. LG41, a fungal endophyte associated with the Chinese medical plant *Xanthium sibiricum*, showed potent antibacterial activity against *S. aureus*, with a MIC of 1 µg mL^−1^ [86]. This result was on par with the antibiotic gentamycin while also showing greater efficacy than streptomycin, which had MIC values of 1 and 5 µg mL^−1^, respectively [86].

#### 2.2.2. Alkaloids and Other Nitrogenous Compounds

**Figure 2:**
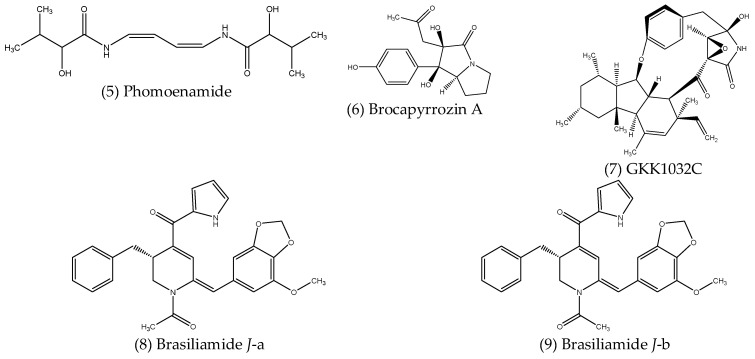
Structures of antimicrobial alkaloids and other nitrogenous compounds produced by endophytes.

Alkaloids are a large class of organic compounds which contain nitrogen atoms in their structure, resulting in molecules with alkaline properties. While plants are well known for their production of many different alkaloids, endophytes also produce these as well as other nitrogen-containing compounds, many of which possess antimicrobial activities. Their structures are shown below in Figure 2. 

Phomoenamide (5), a new enamide dimer isolated from *Phomopsis* sp. PSU-D15, an endophytic fungus obtained from the leaves of *Garcinia dulcis* (Roxb.) Kurz, displayed antimycobacterial activity against *Mycobacterium tuberculosis* (H37Ra strain), with a MIC value of 6.25 µg mL^−1^ [87]. Meng et al. (2017) [88] described twelve alkaloid compounds isolated from the mangrove-associated endophytic fungus *Penicillium brocae* MA-231, six of which were previously unknown. Of these, a new *p*-hydroxyphenopyrrozin derivative, named brocapyrrozin A (6), exhibited significant antibacterial activity against *S. aureus*, with a MIC value of 0.125 µg mL^−1^, which is superior to the positive control chloromycetin, which had a MIC of 4 µg mL^−1^. The same compound also showed potent antifungal activity against *Fusarium oxysporum*, again outperforming the positive control zeocin, with MIC values of 0.25 and 0.5 µg mL^−1^, respectively [88]. A new alkaloid named GKK1032C (7), produced by the mangrove-associated fungal endophyte *Penicillium* sp. CPCC 400817 was reported to have greater antibacterial activity than the antibiotic vancomycin when tested against a strain of MRSA, with MIC values of 1.6 and 2 µg mL^−1^, respectively [89]. Brasiliamide *J* was obtained from the ethyl acetate extracts of *Penicillium janthinellum*, an endophytic fungus isolated from *Panax notoginseng* [90]. Due to the rotation of an amide bond when in solution, Brasiliamide *J* forms two conformers, Brasiliamide *J*-a (8) and Brasiliamide *J*-b (9), with an approximate ratio of 1:2, respectively. Antimicrobial screening of Brasiliamide *J* revealed significant inhibition of the Gram-positive *B. subtilis* and *S. aureus*, with MIC values of 15 and 18 µg mL^−1^, respectively, with no significant inhibition of Gram-negative species. Subsequent investigations showed that treatment with Brasiliamide *J* resulted in morphological changes, causing the cells of *B. subtilis* to grow into longer filaments and *S. aureus* cells to swell by up to two-fold in both cases. Inhibition of the filamentous temperature-sensitive protein Z (FtsZ) was suggested as a potential mechanism for the observed effects, supported by molecular docking results, which showed higher binding energy of Brasiliamide *J* with FtsZ compared to its native ligand [90].

#### 2.2.3. Peptides

**Figure 3:**
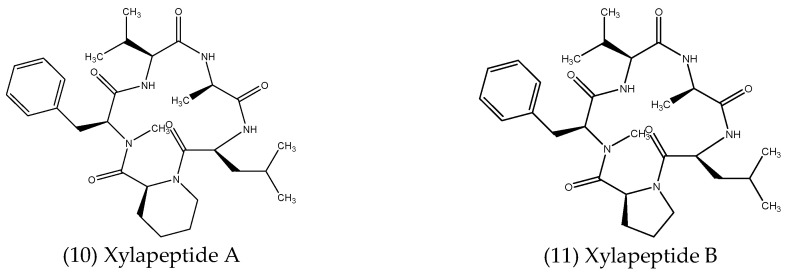
Structures of antimicrobial peptides isolated from endophytes.

Non-ribosomal peptides (NRPs) are a class of structurally diverse secondary metabolites found in fungi and bacteria, synthesised independently of ribosomes by multi-modular enzymes known as non-ribosomal peptide synthetases (NRPSs). NRPs have wide-ranging pharmaceutical applications, including as antitumors, immunosuppressants and antimicrobial compounds, for example [91]. In fact, the first recognised antibiotic, penicillin, is a classic example of an NRP. Numerous peptides have been isolated from endophytes, some of which were reported to possess antimicrobial activities and are described below, with structures shown in Figure 3.

Wei-Feng et al. (2017) [92] described two new cyclopentapeptides isolated from *Xylaria* sp., an endophytic fungus associated with *Sophora tonkinensis*. The first, named xylapeptide A (10), contains the non-proteinogenic amino acid, *L*-pipecolinic acid (*L*-Pip), the first of its kind from a terrestrial source, while the second, named xylapeptide B (11), contains a proline (*L*-Pro) subunit in place of *L*-Pip, this being the only difference between the two compounds. Antibacterial assays revealed that xylapeptide A displayed selective inhibitory effects against *B. cereus* and *B. subtilis*, with a MIC value of 12.5 µg mL^−1^ against each. Xylapeptide B, however, displayed a much broader antimicrobial spectrum, inhibiting *B. cereus* and *Bacillus megaterium* at a MIC of 6.25 µg mL^−1^, while a MIC value of 12.5 µg mL^−1^ was reported against several bacterial pathogens, including *B. subtilis*, *M. luteus*, *S. aureus* and *Shigella castellani*, as well as the fungal pathogen *C. albicans*, suggesting different roles for the *L*-Pip and *L*-Pro residues in the observed antimicrobial effects [92]. 

#### 2.2.4. Phenolic Compounds

**Figure 4:**
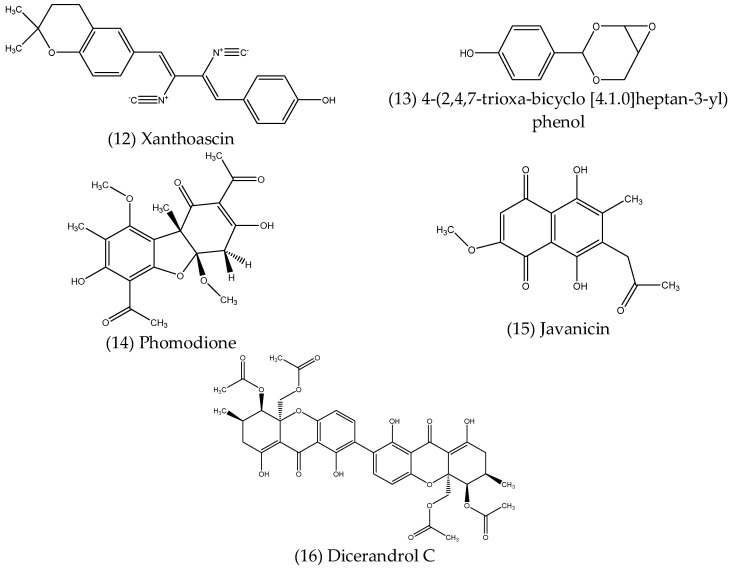
Structures of antimicrobial phenolic compounds produced by endophytes.

Phenolics are a large and diverse class of organic compounds, characterised by the presence of an aromatic ring with at least one bound hydroxyl group within their structure. They are further classified broadly as phenolic acids and polyphenols, which contain one or more aromatic rings, respectively. Phenolic compounds of many types are widely produced as plant secondary metabolites and are well known for their bioactive properties [93]. Endophytes have also been found to produce phenolic compounds, some of which display antimicrobial activities against various pathogens and are described below. The structures of these compounds are shown in Figure 4.

Xanthoascin (12), isolated from the culture of *Aspergillus* sp. IFB-YXS, a fungal endophyte obtained from leaves of *Ginkgo biloba* L., exhibited significant inhibitory activity against the phytopathogenic bacterium *Clavibacter michiganense* subsp. *sepedonicus*, outperforming the antibiotic streptomycin used as a positive control for the assay, for which the MIC values were 0.31 and 0.62 µg mL^−1^, respectively [94]. A preliminary investigation into the mechanism for the observed antibacterial action revealed that xanthoascin treatment resulted in altered cell permeability and subsequent leakage of nucleic acids from the cytomembrane [94]. Subban, Subramani and Johnpaul (2013) [95] reported the isolation of a novel phenolic, 4-(2,4,7-trioxa-bicyclo [4.1.0] heptan-3-yl) phenol (13), from *Pestalotiopsis mangiferae*, a fungal endophyte obtained from the leaves of *Mangifera indica* Linn. The newly described compound displayed strong antibacterial activity against several bacterial pathogens, including *B. subtilis* and *K. pneumoniae* (MIC = 0.039 µg mL^−1^), *E. coli* and *M. luteus* (MIC = 1.25 µg mL^−1^) and *P. aeruginosa* (MIC = 5.0 µg mL^−1^), as well as potent antifungal activity against *C. albicans* (MIC = 0.039 µg mL^−1^), in each case performing better than the respective positive controls, gentamycin and nystatin. The mechanism of antibacterial action against *E. coli*, *P. aeruginosa* and *K. pneumoniae*, was investigated by transmission electron microscope (TEM) analysis, which revealed the agglutination of cytoplasm with pore formation occurring in cell walls resulted in the destruction of bacterial cells [95]. A new usnic acid derivative, phomodione (14), isolated from an endophytic *Phoma* sp. obtained from *Saurauia scaberrinae*, was found to have significant antibacterial activity against *S. aureus* (MIC = 1.6 µg mL^−1^), and also antifungal activity against the oomycete *Pythium ultimum*, the ascomycete *Sclerotinia sclerotiorum* and the basidiomycete *Rhizoctonia solani*, with MIC values of 4–5, 3–5 and 5–8 µg mL^−1^, respectively [96]. Javanicin (15), a highly functionalised naphthaquinone, was isolated from *Chloridium* sp., a fungal endophyte of *Azadirachta indica* A. Juss. [97]. While javanicin displayed only moderate activity against various fungal and bacterial pathogens, such as *C. albicans*, *F. oxysporum*, *E. coli* and a *Bacillus* sp., with MIC values ranging from 20–40 µg mL^−1^, *Pseudomonas* sp., including *P. aeruginosa* and *Pseudomonas fluorescens*, were much more susceptible, with MIC values of 2 µg mL^−1^ in each case, suggesting javanicin could potentially be used as a selective treatment for diseases caused by bacteria of this genus [97]. The polyphenolic compound, dicerandrol C (16), isolated from *Phomopsis longicolla*, an endophytic fungus associated with the red seaweed *Bostrychia radicans*, exhibited significant antibacterial activity against *S. aureus* ATCC 6538 and *Staphylococcus saprophyticus* ATCC 15305, with MIC values of 1 and 2 µg mL^−1^, respectively [98].

#### 2.2.5. Polyketides

**Figure 5:**
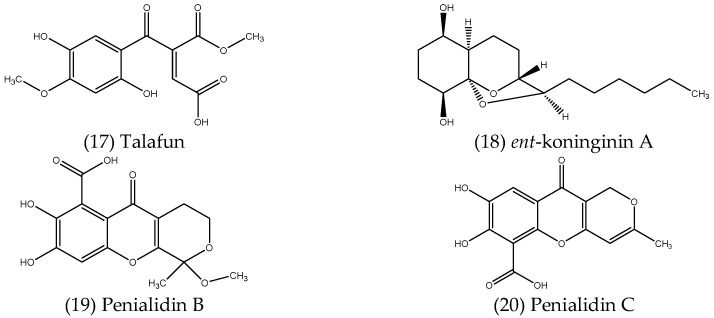
Structures of antimicrobial polyketides isolated from endophytes.

Polyketides are a large group of natural metabolites containing multiple β-hydroxyketone or β-hydroxyaldehyde groups, produced by diverse enzymatic complexes called polyketide synthases (PKSs). Polyketides are structurally diverse and are synthesised from coenzyme-A fatty esters derived from acetate or other short-chain fatty acids, such as propionate or butyrate, and have been found to exhibit a wide range of bioactivities [99]. Several antimicrobial polyketides isolated from endophytes have been reported in the literature, some examples of which are described below, structures of which are shown in Figure 5.

Talafun (17), a new polyketide compound isolated from *Talaromyces funiculosus*, an endophytic fungus obtained from *Salicornia bigelovii*, displayed selective antibacterial activity against *E. coli*, with a MIC value lower than that of ampicillin, which was 18 ± 0.4 and 22 ± 0.4 µM, respectively [100]. Shi et al. (2017) [101] described five new polyketides, along with two known analogues, isolated from the culture extracts of *Trichoderma koningiopsis* QA-3, a fungal endophyte associated with *Artemisia argyi*. Among the isolated compounds, a new tricyclic polyketide with an octahydrochromene framework, named *ent*-koninginin A (18), was found to possess significant antibacterial activity against the marine pathogenic bacteria *Vibrio anguillarum* and *Vibrio vulnificus*, with MIC values of 8 and 4 µg mL^−1^, respectively, as well as antifungal activity against several plant-pathogenic fungi, including *Ceratobasidium cornigerum* (MIC = 8 µg mL^−1^), *Physalospora piricola* Nose. (MIC = 16 µg mL^−1^) and *Penicillium digitatum* (MIC = 32 µg mL^−1^) [101]. Two new polyketides, penialidins B (19) and C (20), were isolated from *Penicillium* sp., an endophytic fungus obtained from the leaves of *Garcinia nobilis* [102]. Penialidin C exhibited strong antibacterial activity against *S. aureus* and *B. subtilis*, with MIC values of 5 µg mL^−1^ in both cases, the same as the MIC of the antibiotic streptomycin used as a positive control and was also active against *E. coli* at 10 µg mL^−1^. Penialidin B was active against *S. aureus* and *E. coli*, each with MIC values of 10 µg mL^−1^ [102].

#### 2.2.6. Terpenoid Compounds

**Figure 6:**
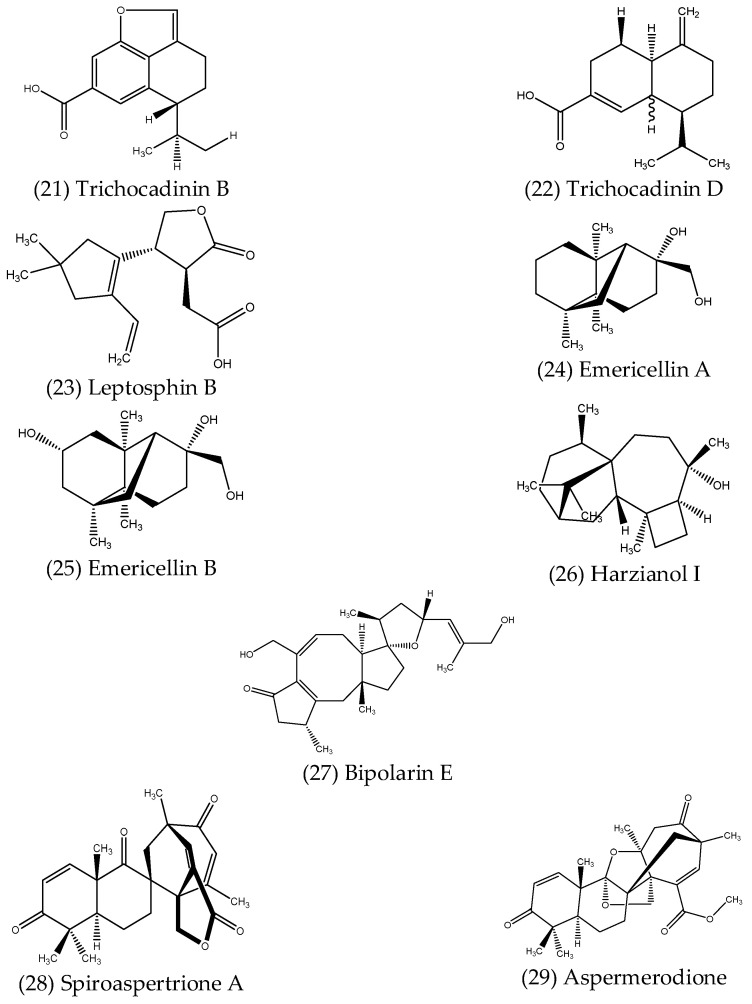
Structures of antimicrobial terpenoids produced by endophytes.

Terpenes and terpenoids represent the largest class of natural products, comprising one or more 5-carbon isoprene units. While terpenes are hydrocarbons, terpenoids are those which also have oxygen within their structure. The terpenoids are further characterised according to the number of isoprene units they contain, such as monoterpenoids (2 isoprene units), sesquiterpenoids (3 isoprene units), diterpenoids (4 isoprene units), sesterpenoids (5 isoprene units) and so on. Many terpenoids possess diverse biological activities, and those produced by endophytes are no exception. Examples of antimicrobial terpenoid compounds isolated from endophytes are described below, along with their reported activities, with structures shown in Figure 6.

Shi et al. (2019) [103] reported six new cadinene-type sesquiterpene derivatives isolated from *Trichoderma virens* QA-8, a fungal endophyte associated with *Artemisia argyi*. Of these, trichocadinin B (21) and trichocadinin D (22) displayed significant antibacterial activity against a broad range of bacterial species, with MIC values of these two compounds against *E. coli*, *Aeromonas hydrophila*, *P. aeruginosa*, *Vibrio harveyi* and *Vibrio parahaemolyticus* ranging from 4–8 and 2–8 µg mL^−1^, respectively. Trichocadinin B also exhibited significant antifungal activity against several plant-pathogenic fungi, with MIC values close to those of the positive control, amphotericin B, in many cases [103]. The new sesquiterpenoid, leptosphin B (23), isolated from *Leptosphaeria* sp. XL026, an endophytic fungus obtained from the leaves of *P. notoginseng*, showed moderate antibacterial activity against *B. cereus*, with a MIC of 12.5 µg mL^−1^ [104]. Pang et al. (2018) [105] described two novel sesquiterpenoids, emericellins A (24) and B (25), isolated from *Emericella* sp. XL 029, a fungal endophyte also obtained from the leaves of *P. notoginseng*. Interestingly, each of these compounds feature an unprecedented tricyclo [4.4.2.1] hendecane scaffold, representing a new type of sesquiterpenoid skeleton. Both displayed minor antibacterial activity against *B. subtilis*, *B. cereus* and *E. coli*, with MIC values between 25–50 µg mL^−1^, as well as antifungal activity against *Verticillium dahliae*, *Helminthosporium maydis* and *Botryosphaeria berengeriana*, with MIC values again ranging from 25–50 µg mL^−1^ [105]. Several new harziane diterpenoids containing a rare and complex 4/7/5/6 tetracyclic scaffold were isolated from *Trichoderma atroviride*, an endophytic fungus obtained from the flower of *Colquhounia coccinea* var. *mollis* [106]. One of the isolated diterpenoids, harzianol I (26), displayed significant antibacterial activity against *S. aureus*, *B. subtilis* and *M. luteus*, with calculated half-maximal effective concentrations (EC_50_) of 7.7, 7.7 and 9.9 µg mL^−1^, respectively [106]. Liu et al. (2019) [107] described bipolarin E (27), which showed strong antibacterial activity against *E. faecalis* (MIC = 8 µg mL^−1^), as well as weaker activity against *P. aeruginosa* (MIC = 32 µg mL^−1^). This compound was one of eight new ophiobolin-type sesterterpenes isolated from the wheat-associated fungal endophyte *Bipolaris* sp. TJ403-B1, all of which feature a rare oxaspiro [4,4] nonane moiety [107]. Meroterpenoids are a group of hybrid terpenoid compounds that are derived from mixed biosynthetic pathways. Two novel antibacterial terpene-polyketide hybrids were isolated from *Aspergillus* sp. TJ23, an endophytic fungus obtained from the leaves of *Hypericum perforatum* [108,109]. The first, spiroaspertrione A (28), a spiromeroterpenoid which contains a unique spiro [bicyclo [3.2.2] nonane-2,1′-cyclohexane] carbocyclic skeleton, exhibited significant antibacterial activity against MRSA, with a MIC value of 4 µg mL^−1^ [108]. Additionally, spiroaspertrione A was also found to work synergistically with oxacillin, reducing the MIC up to 32-fold (from 32 to 1 µg mL^−1^) [108]. Interestingly, according to the Clinical and Laboratory Standards Institute (CLSI), the susceptibility breakpoint for oxacillin against MRSA is ≤2 µg mL^−1^ [110], meaning the co-application of spiroaspertrione A and oxacillin effectively resensitised MRSA to this antibiotic. The second terpene-polyketide hybrid, aspermerodione (29), which contains an unusual 2,6-dioxabicyclo [2.2.1] heptane core, was found to inhibit MRSA, with a MIC value of 32 µg mL^−1^, and also worked synergistically with the β-lactam antibiotics oxacillin and piperacillin, reducing their MIC values by 8- and 16-fold, respectively [109]. In both cases, the molecular docking investigations revealed that these compounds act as potential inhibitors of the penicillin-binding protein 2a (PBP2a), resulting in the synergistic activity with β-lactam antibiotics [108,109].

**Table 2:** List of newly discovered antimicrobial compounds isolated from endophytic fungi.

Chemical Class	Endophyte	Host Plant	Activity	Reference
Aliphatic compounds	*Curvularia papendorfii*	*Vernonia amygdalina*	Antibacterial	[84]
	*Pestalotiopsis fici*	Unidentified	Antifungal	[85]
	*Eupenicillium* sp. LG41	*Xanthium sibiricum*	Antibacterial	[86]
Alkaloids	*Phomopsis* sp. PSU-D15	*Garcinia dulcis* (Roxb.)	Antimycobacterial	[87]
	*Penicillium brocae* MA-231	Mangroves	Antibacterial Antifungal	[88]
	*Penicillium* sp. CPCC 400817	Mangroves	Antibacterial	[89]
	*Penicillium janthinellum*	*Panax notoginseng*	Antibacterial	[90]
Peptides	*Xylaria* sp.	*Sophora tonkinensis*	Antibacterial Antifungal	[92]
Phenolics	*Aspergillus* sp. IFB-YXS	*Ginkgo biloba* L.	Antibacterial	[94]
	*Pestalotiopsis mangiferae*	*Mangifera indica* Linn.	Antibacterial Antifungal	[95]
	*Phoma* sp.	*Saurauia scaberrinae*	Antibacterial Antifungal	[96]
	*Chloridium* sp.	*Azadirachta indica* A. Juss.	Antibacterial	[97]
	*Phomopsis longicolla*	*Bostrychia radicans*	Antibacterial	[98]
Polyketides	*Talaromyces funiculosus*	*Salicornia bigelovii*	Antibacterial	[100]
	*Trichoderma koningiopsis* QA-3	*Artemisia argyi*	Antibacterial Antifungal	[101]
	*Penicillium* sp.	*Garcinia nobilis*	Antibacterial	[102]
Terpenoids	*Trichoderma virens* QA-8	*Artemisia argyi*	Antibacterial Antifungal	[103]
	*Leptosphaeria* sp. XL026	*P. notoginseng*	Antibacterial	[104]
	*Emericella* sp. XL 029	*P. notoginseng*	Antibacterial Antifungal	[105]
	*Trichoderma atroviride*	*Colquhounia coccinea* var. *mollis*	Antibacterial	[107]
	*Bipolaris* sp. TJ403-B1	Wheat	Antibacterial	[107]
	*Aspergillus* sp. TJ23	*Hypericum perforatum*	Antibacterial	[108,109]

### 2.3. Antibiofilm Compounds Produced by Endophytic Fungi

The antimicrobial compounds described so far are effective against planktonic or free-living bacterial cells; however, most bacterial species favour the biofilm mode of growth in natural environments. Compared to the planktonic bacterial cells, those living within biofilms are highly resistant to immune system responses and antibiotic treatment, making them difficult or, at times, impossible to treat. Microbial biofilms are a major cause of infections associated with medical implants and commonly affect areas of the body, such as the urinary tract, lungs and wounds, resulting in persistent infections [111]. It has been estimated that biofilms may be involved in more than 80 % of microbial infections occurring in developed countries [111]. Given the challenges involved in treating biofilm-associated infections, there has been growing interest in compounds that can effectively prevent or eradicate microbial biofilms. Many natural products have been reported to interfere with various stages of biofilm formation and inhibit their development, while recent evidence has shown that some endophyte secondary metabolites also possess such properties.

Meenambiga and Rajagopal (2018) [112] reported that the chloroform and ethyl acetate extracts of *Aspergillus nidulans*, a fungal endophyte associated with the medicinal plant *Acacia nilotica*, inhibited biofilm formation by an oral pathogenic strain of *C. albicans* by 74.86% and 72.53%, respectively, with molecular docking studies suggesting the reduction in virulence was associated with good binding interactions between several compounds present in the extracts and N-myristoyltransferase (NMT), an enzyme involved in growth regulation and various other biological processes [112]. Another factor known to play an important role in the biofilm formation of a variety of bacterial species is quorum sensing; hence, compounds that can interfere with this process also have the potential as antibiofilm agents. Rashmi et al. (2018) [113] showed that the crude extracts obtained from *A. alternata*, an endophytic fungus isolated from *Carica papaya*, were able to affect the quorum-sensing ability of *P. aeruginosa*. Treatment with the extract resulted in the inhibition of several quorum sensing-associated factors, including pyocyanin production (64.6%) and chitinase activity (83.2%), as well as rhamnolipid production and protease activity. The extract was also shown to inhibit biofilm formation by up to 65.2%, while key factors involved in biofilm production were also inhibited, including exopolysaccharide (EPS) production (72.2%) and cell-surface hydrophobicity (CSH) (71.6%), indicating the great potential of endophyte natural products as an alternative to conventional therapeutics for the treatment of *P. aeruginosa* infections [113].

**Figure 7:**
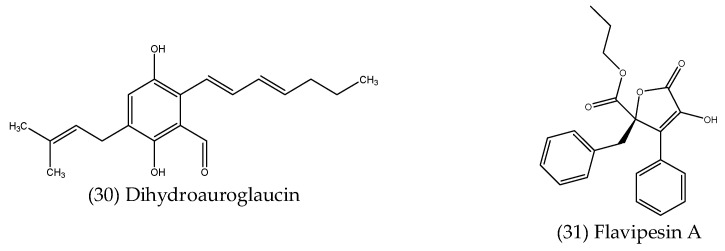
Structures of antibiofilm compounds produced by endophytes.

Other compounds produced by endophytes can disrupt established biofilms by interfering with or penetrating the biofilm matrix. Such compounds have the potential to be used alone in the treatment of biofilm-related infections or in combination with existing antibiotics, as it would allow the antibiotic to reach the microbial cells embedded in the biofilm. Fathallah et al. (2019) [114] isolated dihydroauroglaucin (30) (Figure 7), a prenylated benzaldehyde derivative from *Aspergillus amstelodami*, an endophytic fungus associated with the fruits of *Ammi majus*. Dihydroauroglaucin was found to affect the established biofilms of several test species, with minimum biofilm inhibition concentrations (MBIC) of 7.81 µg mL^−1^ against *S. aureus* and *E. coli*, 15.63 µg mL^−1^ against *Streptococcus mutans* and the yeast *C. albicans*, and 31.25 µg mL^−1^ against *P. aeruginosa* [114]. A new aromatic butyrolactone, flavipesin A (31) (Figure 7), isolated from *Aspergillus flavipes*, a fungal endophyte obtained from the mangrove plant *Acanthus ilicifolius*, was found to decrease the number of living cells within an established *S. aureus* biofilm at concentrations ranging from 390.6–97.7 µg mL^−1^, indicating this compound was able to penetrate the biofilm matrix, killing the bacterial cells inside [115].

## 3. Conclusions

At present, our ability to prevent and treat infectious diseases has been highly compromised. As our existing antimicrobial drugs become increasingly ineffective, it is clear that there is a desperate need to find alternative solutions. Endophytes represent one of the most promising alternatives since they have demonstrated a vast capacity for producing novel bioactive metabolites with not only antimicrobial properties but with wide-ranging bioactivities. For this reason, endophytes have grabbed the attention of natural product researchers around the globe, leading to a constant flow of studies reporting the isolation of novel bioactive compounds from endophytes. However, the research conducted has been limited to date, while the ubiquitous nature of endophytes suggests we have barely scratched the surface. Much greater effort is required to accelerate the research and development of endophytic bioactive compounds, many of which have already shown their promise for treating a wide range of diseases. Altogether, endophytes represent a largely untapped resource for the discovery of novel bioactive compounds with potential applications in drug discovery processes.

## Figures and Tables

**Table 1:** List of crude extracts obtained from endophytes that exhibit antimicrobial activities.

Host Plant	Endophyte	Extract	Active Against	Reference
*Smallanthus sonchifolius*	*Papulaspora immersa*, *Apiospora montagnei* Sacc.	Ethyl acetate, *n*-butanol and water	*E. coli*, *K. rhizophila*, *P. aeruginosa* and *S. aureus*	[72]
*Vitex negundo* L.	*Colletotrichum gloeosporioides*	Methanol	*B. subtilis*, *C. albicans*, *E. coli*, *P. aeruginosa* and *S. aureus* (including several strains of MRSA)	[73]
*Dendrobium devonianum* and *Dendrobium thyrsiflorum*	*Epicoccum nigrum*, *Fusarium tricinctum*, *Phoma* sp.	Ethanol	*B. subtilis*, *C. albicans*, *E. coli* and *S. aureus*	[74]
*Aloe vera*	*Talaromyces wortmannii*	Ethyl acetate	*E. faecalis*, MRSA, *P.acnes*, *S. epidermidis* and *S. pneumoniae*	[75]
*Anemone tomentosa*	Unidentified	Ethyl acetate, *n*-butanol and methanol	*B. licheniformis*, *E. coli*, *K. pneumoniae*, *P. aeruginosa*, *S. aureus* and *S. uberis*	[76]
*Schinus terebinthifolius*	*Alternaria* sp., *Bjerkandera* sp., *Diaporthe* sp., *Penicillium* sp., *Xylaria* sp.	Ethyl acetate and methanol	*C. albicans*, *P. aeruginosa* and *S. aureus*	[77]
*Mirabilis jalapa* L.	*Aspergillus clavatonanicus*	Ethyl acetate	*B.subtilis*, *E. coli*, *M. luteus* and *S. aureus*	[78]
*Ocimum basilicum* var. *thyrsiflora*	*Nigrospora* sp.	Ethyl acetate	*B. cereus*, *E. coli*, *S. epidermidis* and *V. cholerae*	[79]
*Solanum surattense*	*Penicillium roqueforti*, *Trichoderma reesei*	Ethyl acetate	*A. tumefaciens*, *X. oryzae*, *P. syringae* and *R. solanacearum*	[80]
*Bruguiera sexangula*	*Gelasinospora endodonta*	Ethyl acetate	*E. coli*	[81]
